# Editorial: Oral complications in cancer patients

**DOI:** 10.3389/froh.2022.1116885

**Published:** 2023-01-26

**Authors:** Wilfredo Alejandro González-Arriagada, Giulia Ottaviani, David Dean, Giulia Ottaviani, Alan Roger Santos-Silva, Nathaniel S. Treister

**Affiliations:** ^1^ Facultad de Odontología, Universidad de los Andes, Las Condes, Chile; ^2^ Centro de Investigación e Innovación em Biomedicina, Universidad de los Andes, Las Condes, Chile; ^3^ Anatomic Pathology, Lino Rossi Research Center, Department of Biomedical, Surgical and Dental Sciences, Università degli Studi di Milano, Milan, Italy; ^4^ Department of Oral Medicine, Fred Hutchinson Cancer Center, University of Washington, Seattle, WA, United States; ^5^ Department of Medicine, Surgery and Health Sciences, University of Trieste, Trieste, Italy; ^6^ Oral Diagnosis Department, Oral Medicine (Stomatology), Piracicaba Dental School, University of Campinas (UNICAMP), Piracicaba, Brazil; ^7^ Division of Oral Medicine and Dentistry, Brigham and Women’s Hospital and Harvard School of Dental Medicine, Boston, MA, United States

**Keywords:** cancer therapy, chemotherapy, oral complications, oral mucositis, oral health, radiotherapy, xerostomia, immune checkpoint inhibitors

**Editorial on the Research Topic** Oral complications in cancer patients

Cancer is the second leading cause of death worldwide and future projections place it as the leading cause by 2040 (1, 2, Salazar-Gamarra et al.). Current strategies of treatment include surgery, chemotherapy, radiotherapy, cellular therapies—e.g., stem cell transplantation, chimeric antigen receptor (CAR)-T therapy, bone-modifying agents, immune checkpoint inhibitors (ICIs), and others. These therapies, especially in advanced cancers, produce direct and indirect toxicities involving the oral cavity and neighboring regions. For this reason, it is essential to recognize the role that trained dentists provide in the multidisciplinary teams that treat cancer patients (Harris et al.). Roles include comprehensive dental evaluation and treatment to decrease infection risk prior to initiation of cancer therapy (Yong et al.), intra-therapy assessment to mitigate acute oral toxicities, and long-term follow-up posttreatment therapy to diagnose and manage late complications including, in some cases, prosthodontic rehabilitation (Salazar-Gamarra et al.). The inclusion of dentistry in this multidisciplinary approach is highly beneficial to the patient, but is not yet universal (3).

Oral complications associated with cancer therapy are frequent and can be classified as early or late onset. Early, or acute complications, are those that begin during therapy and resolve within 1 month of completion. Acute complications include oral mucositis, dysgeusia, hyposalivation, candidiasis, radiodermatitis, and dysphagia. Late, or chronic complications, develop after completion of therapy and in some cases may be permanent. Chronic complications include hyposalivation, trismus, radiation caries, osteonecrosis, and dysphagia, among others. In addition, head and neck cancers often require surgery to treat the primary tumor and regional metastases (neck dissection), resulting in permanent physical sequela requiring multidisciplinary therapy to address functional and social impacts. This topic was chosen to provide new insights into the epidemiology, pathobiology, impact, and management of oral toxicities in cancer patients with the goal of improving patient quality of life.

Mucositis is the principal dose-dependent oral complication of cancer therapy and may lead to interruption of the treatment. Oral mucositis (OM) may trigger febrile neutropenia and blood stream infection and is also commonly associated with feeding problems and the introduction of enteral nutrition (Zecha et al.). Oral mucositis is associated with increased use of hospital resources, physician and multidisciplinary consultations, and prolonged hospitalization (including treatment in intensive care units), increasing cost of care, and the economic burden to patients, in both private and public health systems across various cancer treatment modalities (4). Photobiomodulation, delivered intraorally and extraorally, has shown promising results in OM, including different management approaches, both preventive and curative (Adnan et al). It has also been reported to prevent severe hyposalivation related to radiation therapy (Gobbo et al).

The etiology of oral mucositis has been linked to the direct effects of chemotherapy and radiation in addition to the effects of microbiological co-infection, including the oral-gut axis microbiome. This suggests that control and treatment of microorganisms could be a novel and successful approach to reduce mucositis severity (Al-Qadami et al.). Changes in the microbiome of the oral cavity are related to alteration in saliva, and probiotics have been proposed as an alternative to reduce circulating bacteria and candida in the oral environment (Pispero et al.).

Chronic graft-versus-host disease (GVHD) can broadly impact the oral cavity and oral function in patients undergoing allogeneic hematopoietic cell transplantation. Manifestations include lichenoid mucosal inflammation, lymphocyte-mediated salivary gland dysfunction and associated dental caries, taste and smell disturbances, and trismus. Patients with chronic GVHD are also at increased risk for oral cavity second primary tumors, particularly oral squamous cell carcinoma (Dean and Sroussi and Boor et al.). Immune checkpoint inhibitors have been associated with similar immune-mediated oral toxicities which are still being characterized (Klein et al.).

Salivary gland dysfunction is a potentially permanent side effect of multiple cancer therapies, including head and neck radiation therapy, chronic GVHD, and ICIs. Hyposalivation contributes to the development of caries, candidiasis, and psychological complications related to difficulties in nutrition and social interaction (Vistoso Monreal et al.). Additionally, candidiasis is a common opportunistic infection in cancer patients, secondary to hyposalivation and changes in the quality of saliva.

Radiation caries is a frequent complication of head and neck cancer therapy, characterized rapid onset and destruction of dentition when not promptly diagnosed and treated. Radiation caries can lead to pain, infection, and compromised function. Resulting dental extractions are associated with increased risk of osteoradionecrosis (ORN), which may require extensive surgical resection (Vistoso Monreal et al.). Dental restorations in cancer patients have been shown to have reduced longevity, however, this has yet to yield technological advancement in dental adhesives, resins, and other materials specially designed for patients treated with head and neck radiotherapy (Pedroso et al.). A similar pattern of rampant caries can be observed in GVHD patients. Limited opening secondary to trismus can impede oral hygiene and dental follow-up. Currently, physiotherapy is the first option, but the results are vague and uncertain, giving space to the introduction of surgical alternatives (Smeets et al.).

Osteonecrosis of the jaw can be one of the most impactful late complications, particularly in more extensive cases. Medication-related osteonecrosis of the jaw (MRONJ), like ORN, can be very challenging to manage and may require aggressive surgical resection (Singh et al.). MRONJ is characterized by the exposed necrotic bone and may be related to drug therapy, including antiresorptive and antiangiogenic targeted therapies (Migliorati). Conservative therapy is favored as the first-line intervention and may include irrigation with chlorhexidine, sequestrectomy, and pharmacological coverage with systemic antibiotics and pentoxifylline and tocopherol (Migliorati and Singh et al.). This condition is clinically like ORN, but the differences in etiology and risk factors may affect its treatment and prognosis.

Strategies for risk prediction of oral toxicities related to cancer therapies are needed for a personalized prevention protocol (Sonis) and they have been primarily used in OM. Artificial intelligence and machine learning approaches have been proposed for risk prediction of toxicity for cancer therapy in patients with head and neck cancer (Fanizzi et al.). These strategies have great benefits for the patients and oncologic services because the use of resources is most efficient and effective, reducing the high costs of prevention and treatment of collateral effects.

The rapid evolution of oncologic therapies requires specialists to constantly update themselves to respond to the requirements of patients and their services. It is important to draw attention to the fact that many clinical trials report oral complications in a superficial and protocol-directed manner. We believe that these toxicities need to be considered during the study design stage, including oral medicine expertise within the research team, in order to best characterize these conditions.

This Research Topic provides a glimpse into this complex and ever-evolving oncologic realm of clinical oral medicine.
